# Supplementary material for: Repurposing caspofungin as a small-molecule inhibitor of Clostridium perfringens α-toxin for treatment of gas gangrene
Source: Commun Med (Lond). 2026 Apr 16;6:225. doi: 10.1038/s43856-026-01503-y (PMC13086959; doi:10.1038/s43856-026-01503-y)
Supplement: Supplementary file 1 — Supplementary information [file 43856_2026_1503_MOESM1_ESM.pdf]

Supplementary information for

Repurposing caspofungin as a small-molecule inhibitor of *Clostridium perfringens* α-Toxin for treatment of gas gangrene

Masaya Takehara[1,2,*], Yuta Homma[1], Tomoaki Ishihara[3], Yoshihiko Sakaguchi[2], Yusuke Kasai[4], Kanako Matsumoto[2], Katsuyuki Nakashima[5], Toshiyuki Yamaji[1], Yoshiyuki Tanaka[5], Hiroshi Imagawa[4] & Masahiro Nagahama[2]

[1]Department of Microbiology and Immunology, Faculty of Pharmacy, Juntendo University, 6-8-1 Hinode, Urayasu, Chiba 279-0013, Japan

[2]Department of Microbiology, Faculty of Pharmaceutical Sciences, Tokushima Bunri University, 180 Nishihamabouji Yamashiro-cho, Tokushima 770-8514, Japan

[3]Department of Pharmacy, Faculty of Pharmaceutical Sciences, Nagasaki International University, 2825-7 Huis Ten Bosch, Sasebo, Nagasaki 859-3298, Japan

[4]Chemistry of Functional Molecule, Faculty of Pharmaceutical Sciences, Tokushima

Bunri University, 180 Nishihamabouji Yamashiro-cho, Tokushima 770-8514, Japan

[5]Laboratory of Analytical Chemistry, Faculty of Pharmaceutical Sciences, Tokushima Bunri University, 180 Nishihamabouji Yamashiro-cho, Tokushima 770-8514, Japan

*Correspondence to: Dr. Masaya Takehara, Department of Microbiology and Immunology, Faculty of Pharmacy, Juntendo University, 6-8-1 Hinode, Urayasu, Chiba 279-0013, Japan; E-mail: m.takehara.du@juntendo.ac.jp; Tel.: +81-47-354-3311; Fax: +81-47-381-1141.

Table of Contents

Supplementary methods

Supplementary Fig. 1-4

Supplementary references

Supplementary methods

Titration experiments using circular dichroism (CD) spectroscopy and determination of dissociation constants

CD spectra were recorded in a basal solution containing 10 mM Na_2HPO_4, 20 mM NaCl (pH7.4), and 1.1–2.2 μM α-toxin protein. The basal solutions were titrated with caspofungin or micafungin at the following molar ratios of ligand to α-toxin: for caspofungin/α-toxin, 0.18, 0.36, 0.54, 0.90, 1.2, 2.3, 3.4, 6.9, and 10.3; and for micafungin/α-toxin, 0.18, 0.36, 0.54, 0.72, 1.2, 1.8, 3.5, 5.2, and 10.5. At each titration point, the mixtures were equilibrated at 5 °C for 15 min.

CD spectra were recorded using a J-1100 CD spectrometer equipped with a Peltier-type temperature controller (JASCO Corporation, Tokyo, Japan). Each spectrum was recorded over the wavelength range of 250–200 nm at 0.1 nm intervals, and 16 scans were averaged at each titration point. A baseline spectrum of the basal solution was recorded under identical conditions. The obtained CD spectra were subsequently subjected to least-squares fitting.

The baseline spectrum was subtracted from each titration spectrum. For each ligand, CD intensities were extracted at 5 nm intervals within the 250–200 nm range and used for least-squares fitting. Dissociation constants (K_{d}) were calculated assuming 1:1 complex formation between the α-toxin protein and the ligand. Under this assumption, the theoretical titration curve at a given wavelength is expressed as:

$$\Delta CD = \left(CD_{ini} - CD_{fin}\right)\frac{([P]_0 + [S] + K_{\mathrm{d}}) - \sqrt{([P]_0 + [S] + K_{\mathrm{d}})^2 - 4[P]_0[S]}}{2[P]_0}$$

where ΔCD is the difference in CD intensity at each titration point relative to the initial value, CD_{ini} and CD_{fin} are the theoretical initial and final CD intensities, respectively, $[P]_0$ is the total protein concentration, $[S]$ is the ligand concentration at each titration point, and K_{d} is the dissociation constant. The theoretical CD intensities were fitted to the experimental data using the least-squares method with the Solver add-in in Microsoft Excel (Microsoft, Redmond, WA, USA). In Supplementary Fig. 2, representative titration data of CD intensities at 220 nm are shown for each ligand.

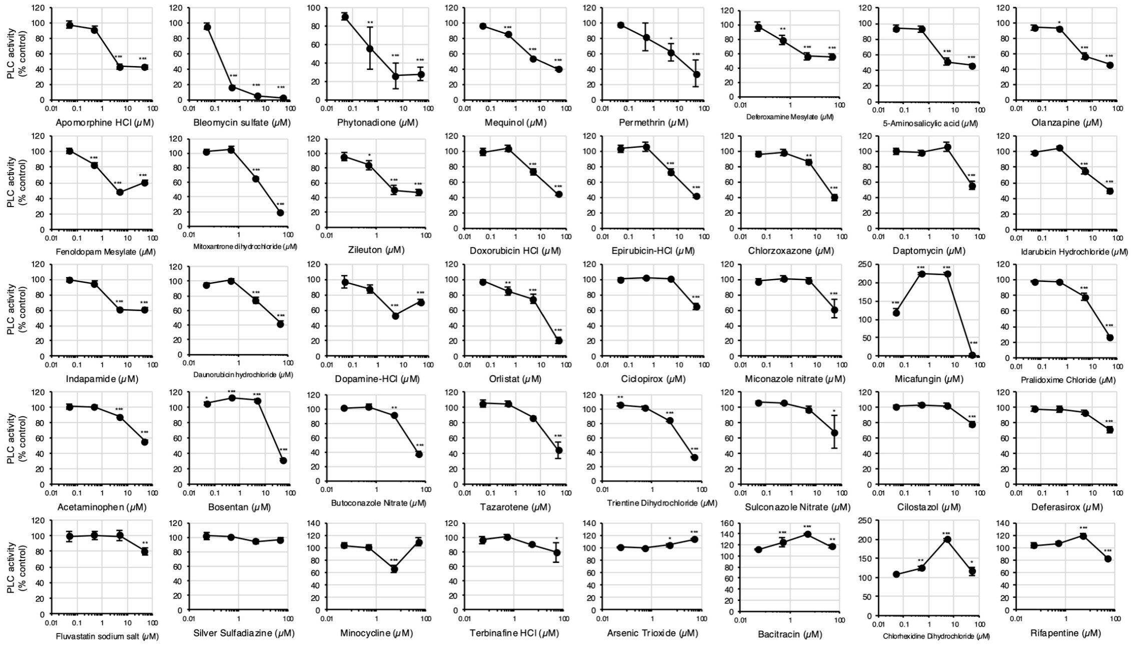

Supplementary Fig. 1 | Inhibitory effects of candidate compounds selected from the primary screening on the PLC activity of α-toxin. The effects of 40 candidate compounds on the PLC activity of 50 ng/ml α-toxin were measured at concentrations ranging from 50 nM to 50 μM. The PLC activity of the toxin was measured using the Amplex red phosphatidylcholine-specific phospholipase C assay kit according to the manufacturer's instructions. The measurement was performed by detecting fluorescence intensity with excitation at 550 nm and emission at 580 nm. A one-way ANOVA was employed to assess significance. Values are the mean ± standard deviation. **P* < 0.05; ***P* < 0.01; ****P* < 0.001.

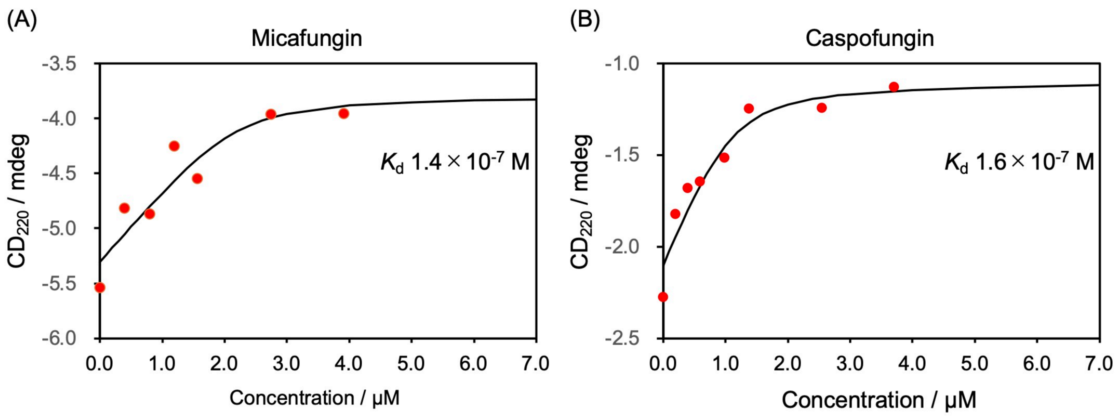

Supplementary Fig. 2 | Titration experiments of α-toxin monitored by CD spectroscopy. (A) CD intensity of α-toxin (2.2 μM) at 220 nm plotted as a function of micafungin concentration at 37 °C. (B) CD intensity of α-toxin (1.1 μM) at 220 nm plotted as a function of caspofungin concentration at 15 °C. In both panels, experimental titration points and fitted theoretical curves are shown as red circles and black lines, respectively. The dissociation constants (K_d) obtained by least-squares fitting are indicated. Due to the limited stability of the α-toxin protein, titration analyses were feasible only under the indicated conditions for each ligand. Because of the difference in experimental temperatures, direct comparison of the K_d values was not possible. Nevertheless, binding of both ligands was evident from the changes in CD intensity.

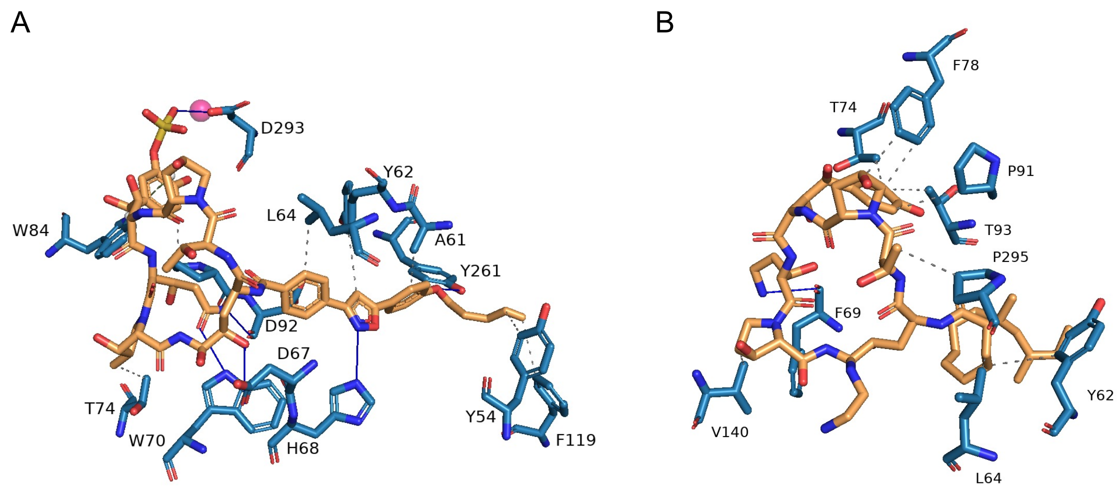

Supplementary Fig. 3 | Interactions of micafungin and caspofungin with α-toxin (PDB ID: 1QM6). Interactions between micafungin (A) or caspofungin (B) and α-toxin (PDB ID: 1QM6) were analyzed using PLIP (Protein-Ligand Interaction Profiler)[34] and visualized using PyMOL[35]. Hydrogen bonds are indicated by blue solid lines, hydrophobic interactions by black dotted lines, π–π stacking interactions by black dashed lines, and metal coordination by a pink sphere.

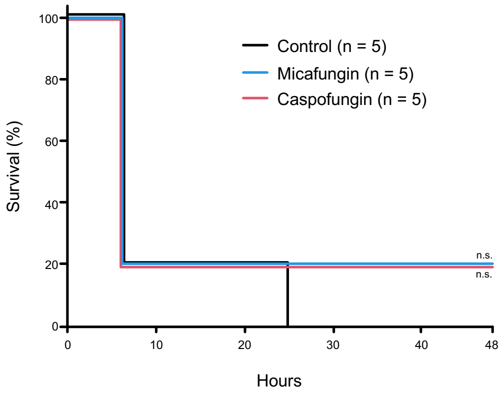

Supplementary Fig. 4 | Assessment of low-dose micafungin and caspofungin on α-toxin-induced lethality. C57BL/6J mice were injected intraperitoneally with 600 ng of α-toxin and 100 μg of micafungin or caspofungin, which had been pre-mixed and diluted in phosphate-buffered saline. The survival of mice was monitored, and Kaplan-Meier survival curves are shown. Log-rank test was employed to assess significance. n.s., not significant.

Supplementary references

34 Salentin, S., Schreiber, S., Haupt, V. J., Adasme, M. F. & Schroeder, M. PLIP: fully automated protein-ligand interaction profiler. *Nucleic Acids Res.* **43**, W443-447 (2015). https://doi.org/10.1093/nar/gkv315

35 The PyMOL Molecular Graphics System, Version 3.1.6.1 Schrödinger, LLC.
