# Supplementary material for: Repurposing caspofungin as a small-molecule inhibitor of Clostridium perfringens α-toxin for treatment of gas gangrene
Source: Commun Med (Lond). 2026 Apr 16;6:225. doi: 10.1038/s43856-026-01503-y (PMC13086959; doi:10.1038/s43856-026-01503-y)
Supplement: Supplementary file 4 — Description of Additional Supplementary Data [file 43856_2026_1503_MOESM4_ESM.docx]

Description of additional supplementary file

File name: Supplementary Data 1

Description: The source data underlying Figs. 1B–D, Figs. 3A–E, Figs. 4A and C, Supplementary Fig. 1, Supplementary Fig. 2, and Supplementary Fig. 4 are provided in Supplementary Data 1.
